# Comparative Efficacy and Safety of Liraglutide Versus Metformin in Women With Polyendocrine Metabolic Ovarian Syndrome (PMOS): A Systematic Review and Meta-Analysis of Randomized Controlled Trials

**DOI:** 10.7759/cureus.115758

**Published:** 2026-09-04

**Authors:** Indrajit Banerjee, Mooroogiah Krissheeven, Nishant Pandey, Shradha Banerjee, Jared Robinson, Indraneel Banerjee

**Affiliations:** 1 Pharmacology, Sir Seewoosagur Ramgoolam Medical College, Belle Rive, MUS; 2 Medicine, Sir Seewoosagur Ramgoolam Medical College, Belle Rive, MUS; 3 Medicine, Manipal College of Medical Sciences, Pokhara, NPL; 4 Research and Development, Royal College Port Louis, Port Louis, MUS; 5 Surgery, Joe Morolong Memorial Hospital, Vryburg, ZAF; 6 Urology, University of Florida Health, Jacksonville, USA

**Keywords:** endocrine disorders, glucagon-like peptide-1 receptor agonist, insulin resistance, liraglutide or saxenda, metformin, pcos and metabolic syndrome, pmos, polycystic ovarian syndrome, polyendocrine metabolic ovarian syndrome, women’s health

## Abstract

Polyendocrine metabolic ovarian syndrome (PMOS), previously known by the name ‘polycystic ovary syndrome (PCOS),' is a common and complex endocrine-metabolic disorder affecting approximately one in eight women. PMOS is increasingly regarded as a multisystem disorder rather than only a gynaecological condition. Exclusive of weight loss, metformin has been the mainstay pharmacological therapy in the treatment of PMOS; a new contender for the management of this condition is liraglutide. Liraglutide is a glucagon-like peptide-1 receptor agonist (GLP-1 RAs) and has gained increasing interest because it targets appetite regulation, body weight, and glycemic control as well as insulin sensitivity. There remains no comprehensive and up-to-date synthesis specifically comparing liraglutide versus metformin across clinically important outcomes in women with PMOS. This systematic review and meta-analysis aims to comprehensively evaluate these outcomes and provide evidence to guide individualized therapeutic decision-making.

This systematic review and meta-analysis followed Preferred Reporting Items for Systematic Reviews and Meta-Analyses 2020 guidelines (PRISMA). The research protocol was registered under the International Prospective Register of Systematic Reviews (PROSPERO), and the proposal is available under registration number CRD420261435182.

No statistically significant difference in BMI between liraglutide and metformin (mean difference (MD)=0.25, 95% confidence interval (CI): -7.30-7.79, P=0.95, I²=82%, p=0.02). The pooled result of Homeostatic Model Assessment for Insulin Resistance (HOMA-IR) favored metformin but showed no statistical difference between liraglutide and metformin (MD=0.94, 95% CI: -0.09-1.97, P=0.07, I²=0%, chi-square=0.14, p=0.71). Although the pooled effect favored metformin due to a higher frequency of the menstrual cycle, there was no significant difference between liraglutide and metformin (MD = -1.42, 95% CI: -4.57-1.73, P = 0.38, I² = 75%). A fixed-effect meta-analysis demonstrated no significant difference in fasting glucose levels between liraglutide and metformin (MD=-0.01 mmol/l, 95 % CI: - 0.82-0.80, P=0.98, I^2^=37%, p=0.21). No significant difference in fasting insulin levels between liraglutide and metformin (MD=-1.11 Mu/L, 95% CI: -6.99-4.77, p=0.71, I²=0%, p=0.69). Pooled analysis demonstrated no significant difference in the risk of nausea (risk ratio (RR)=0.91, 95% CI: 0.53-1.55, P=0.73), although the effect estimate numerically favored liraglutide. The incidence of diarrhea was significantly lower in the liraglutide group compared to metformin. The pooled analysis demonstrated a reduced risk of diarrhea with liraglutide (RR=0.31, 95% CI: 0.12-0.81, P=0.02), suggesting a 69% relative risk reduction as compared to metformin.

The available randomized evidence suggests that liraglutide and metformin have comparable effects on BMI, insulin resistance measured by HOMA-IR, fasting glucose, fasting insulin, and menstrual-cycle frequency in women with PMOS. Although both agents have demonstrated clinical benefits in the management of PMOS, the current evidence does not demonstrate a statistically significant advantage of liraglutide over metformin for the assessed outcomes. However, the absence of a statistically significant difference cannot be interpreted as evidence of therapeutic equivalence or non-inferiority, nor does it exclude a clinically meaningful benefit of either treatment.

## Introduction and background

Polyendocrine metabolic ovarian syndrome (PMOS), previously known as ‘polycystic ovary syndrome (PCOS)', is a common and complex endocrine-metabolic disorder affecting approximately one in eight women and nearly 170 million women of reproductive age worldwide [1,2]. The newer term PMOS has been proposed because the traditional name may incorrectly suggest that ovarian cysts are the main features of the condition, but its manifestations extend far beyond the ovaries and include several metabolic, reproductive, endocrinological, dermatological, and psychological abnormalities [2]. In adults, diagnosis is generally based on the presence of at least two of the following after excluding other relevant disorders: oligo-anovulation, clinical or biochemical hyperandrogenism, and polycystic ovarian morphology on ultrasound or elevated anti-Müllerian hormone levels [1,2].

PMOS is increasingly regarded as a multisystem disorder rather than only a gynecological condition. Its clinical features include irregular menstrual cycles, anovulation, infertility, acne, hirsutism, alopecia, obesity, dyslipidemia, impaired glucose metabolism, type 2 diabetes, hypertension, sleep apnea, and metabolic dysfunction-associated steatotic liver disease [1,2]. Psychological disorders, including anxiety, depression, eating disorders, psychosexual dysfunction, poor body image, and a reduced quality of life, are also common in those suffering from PMOS [1,2]. Furthermore, women with PMOS have a greater risk of pregnancy complications, including miscarriage, gestational diabetes, hypertension, preeclampsia, endometrial hyperplasia, and endometrial cancer, especially in the presence of prolonged untreated amenorrhea, higher body weight, and type 2 diabetes [1,3].

Insulin resistance and compensatory hyperinsulinaemia are considered major contributors to the pathophysiology of PMOS and are present in approximately 50%-80% of affected women [1]. These abnormalities promote androgen excess, ovulatory dysfunction, and an increased metabolic risk. Obesity is also highly prevalent, affecting around 20-80% of patients and can further worsen insulin resistance, endocrine disturbances, menstrual irregularity, and infertility [1]. Lifestyle intervention remains the first-line approach, as even a modest weight loss of 2%-10% may improve endocrine function and restore more regular menstrual cycles [4]. However, lifestyle intervention alone is often difficult to maintain, creating a need for effective pharmacological options.

Metformin is one of the most widely used insulin-sensitizing agents in PMOS. It reduces hepatic gluconeogenesis, improves peripheral glucose utilization, and enhances insulin sensitivity without usually causing weight gain or hypoglycemia [5,6]. Current guidelines recommend metformin particularly for women with a body mass index (BMI) of 25 kg/m² or above to improve metabolic and anthropometric outcomes, although it may also be considered in women with a lower BMI [3]. Evidence suggests that metformin can improve insulin resistance, androgen levels, ovulation, and menstrual frequency, although its effects on weight and menstrual function have not been fully consistent across studies [5,6]. Gastrointestinal adverse effects are common, but they often decrease with continued treatment [6].

Liraglutide, a glucagon-like peptide-1 receptor agonist (GLP-1 RA), has gained increasing interest because it targets appetite regulation, body weight, glycemic control, and insulin sensitivity [3,4]. Available studies suggest that GLP-1 RAs may produce greater reductions in BMI and waist circumference than metformin, and some studies have also reported improvements in menstrual regularity and reproductive hormones [4,5]. Combination therapy with liraglutide and metformin has shown additional benefits for weight loss, waist circumference, and hormonal outcomes compared with metformin alone [5]. Nevertheless, evidence regarding menstrual regulation, reproductive outcomes, and safety remains uncertain [4].

Despite the increasing use of GLP-1 RAs in the management of PCOS, important evidence gaps remain. Previous systematic reviews and meta-analyses have primarily evaluated GLP-1 RAs as a therapeutic class by pooling different agents, including liraglutide, exenatide, and semaglutide, or have combined monotherapy with combination therapy, thereby limiting drug-specific conclusions regarding liraglutide [7-10]. Furthermore, the available evidence is based on a limited number of randomized controlled trials (RCTs) with small sample sizes, predominantly short follow-up periods, considerable clinical and statistical heterogeneity, and generally low or very low certainty of evidence [7-10]. Although improvements in metabolic, anthropometric, and selected hormonal outcomes have been reported, findings regarding comparative efficacy, menstrual cycle regulation, weight outcomes, and particularly the safety profile of liraglutide compared with metformin remain inconsistent or inadequately synthesized across existing reviews [7-10]. Consequently, there remains no comprehensive and up-to-date synthesis specifically comparing liraglutide versus metformin across these clinically important outcomes in women with PMOS. Therefore, the present systematic review and meta-analysis was undertaken to comprehensively evaluate these outcomes and provide evidence to guide individualized therapeutic decision-making.

## Review

Methods

Registration and Protocol

This systematic review and meta-analysis followed the Preferred Reporting Items for Systematic Reviews and Meta-Analyses (PRISMA) 2020 guidelines [11]. The research protocol was registered under the International Prospective Register of Systematic Reviews (PROSPERO) with ID number CRD420261435182. The full protocol is available in PROSPERO [12].

Search Strategy

A thorough search was done on PubMed, Cochrane Central Register of Controlled Trials (CENTRAL), Turning Research into Practice (TRIP), and Google Scholar in order to identify relevant manuscripts from 2014 to 2026. A combination of MeSH/ keywords and Boolean operators was used for data extraction. They are as follows: (((Ovary Syndrome, Polycystic[Title/Abstract] OR Syndrome, Polycystic Ovary[Title/Abstract] OR Polycystic Ovarian Syndrome[Title/Abstract] OR Ovarian Syndrome, Polycystic[Title/Abstract] OR Polycystic Ovary Syndrome 1[Title/Abstract] OR Sclerocystic Ovarian Degeneration[Title/Abstract] OR Ovarian Degeneration, Sclerocystic[Title/Abstract] OR Sclerocystic Ovary Syndrome[Title/Abstract] OR Stein-Leventhal Syndrome[Title/Abstract] OR Stein Leventhal Syndrome[Title/Abstract] OR Syndrome, Stein-Leventhal[Title/Abstract] OR Sclerocystic Ovaries[Title/Abstract] OR Ovary, Sclerocystic[Title/Abstract] OR Sclerocystic Ovary[Title/Abstract] OR (Polyendocrine metabolic ovarian syndrome[Title/Abstract]) OR PMOS[Title/Abstract] OR Polycystic Ovarian Syndrome[Title/Abstract] OR PCOS[Title/Abstract]) OR (Dimethylbiguanidine OR Dimethylguanylguanidine OR Glucophage OR Metformin Hydrochloride OR Hydrochloride, Metformin OR Metformin HCl OR HCl, Metformin OR Metformin)) OR (Liraglutide OR NN 2211 OR NN-2211 OR NN2211 OR Victoza OR Saxenda)) OR (placebo). The strategy employed in the search, along with the total number of articles screened, is presented in Table 1. 

**Table 1:** Search strategy and various databases used Summary of databases searched, keywords/MeSH terms used, search strategies, applied filters, and the number of records retrieved

Date and time of search	Database used	MeSH terms used	Filters used	Total number of manuscripts
30/08/26 at 16:43	PubMed	(((Ovary Syndrome, Polycystic[Title/Abstract] OR Syndrome, Polycystic Ovary[Title/Abstract] OR Polycystic Ovarian Syndrome[Title/Abstract] OR Ovarian Syndrome, Polycystic[Title/Abstract] OR Polycystic Ovary Syndrome 1[Title/Abstract] OR Sclerocystic Ovarian Degeneration[Title/Abstract] OR Ovarian Degeneration, Sclerocystic[Title/Abstract] OR Sclerocystic Ovary Syndrome[Title/Abstract] OR Stein-Leventhal Syndrome[Title/Abstract] OR Stein Leventhal Syndrome[Title/Abstract] OR Syndrome, Stein-Leventhal[Title/Abstract] OR Sclerocystic Ovaries[Title/Abstract] OR Ovary, Sclerocystic[Title/Abstract] OR Sclerocystic Ovary[Title/Abstract] OR (Polyendocrine metabolic ovarian syndrome[Title/Abstract]) OR PMOS[Title/Abstract] OR Polycystic Ovarian Syndrome[Title/Abstract] OR PCOS[Title/Abstract]) OR (Dimethylbiguanidine OR Dimethylguanylguanidine OR Glucophage OR Metformin Hydrochloride OR Hydrochloride, Metformin OR Metformin HCl OR HCl, Metformin OR Metformin)) OR (Liraglutide OR NN 2211 OR NN-2211 OR NN2211 OR Victoza OR Saxenda)) OR (placebo)	RCT, English, Humans, From 2014 to 2026	56727
30/08/26 at 16:50	Cochrane	MeSH descriptor:[Polycystic Ovary Syndrome] explode all trees OR MeSH descriptor:[metformin] explode all trees OR MeSH descriptor:[liraglutide] explode all trees OR MeSH descriptor:[Placebos] explode all trees	From 2014 to 2026	10
30/08/26 at 16:52	Turning Research into Practice (TRIP)	Polyendocrine metabolic ovarian syndrome OR PMOS OR Polycystic Ovarian Syndrome OR PCOS AND Liraglutide OR GLP-1 OR Glucagon like Peptide-1 OR Metformin OR Biguanides OR placebo	From 2014 to 2026	84
30/08/26 at 16:53	Google Scholar	Polyendocrine metabolic ovarian syndrome OR PMOS OR Polycystic Ovarian Syndrome OR PCOS AND Liraglutide OR GLP-1 OR Glucagon like Peptide-1 OR Metformin OR Biguanides OR placebo	-	492
TOTAL				57313

Eligibility Criteria

The eligibility criteria for this systematic review and meta-analysis were defined according to the Population, Intervention, Comparison, Outcomes, Study design (PICOS) framework to ensure a structured and reproducible study selection process. The population comprised patients diagnosed with PMOS. The intervention evaluated was liraglutide in the interventional group compared with metformin in the control group. The outcomes were BMI, Homeostatic Model Assessment for Insulin Resistance (HOMA-IR), frequency of menstrual cycles, glucose levels, insulin levels, and adverse effects. The study design included RCTs comparing liraglutide versus metformin, liraglutide versus placebo, and metformin versus placebo in women with PMOS (Table 2).

**Table 2:** PICOS framework was used to guide study selection and data extraction for this systematic review.

Framework	
Population (P)	Patients diagnosed with polyendocrine metabolic ovarian syndrome
Intervention (I)	Liraglutide, metformin, or placebo
Comparison (C)	Liraglutide versus metformin, liraglutide versus placebo, and metformin versus placebo
Outcome (O)	BMI, Homeostatic Model Assessment for Insulin Resistance, frequency of menstrual cycles, glucose levels, insulin levels, and adverse effects
Study design (S)	Randomized controlled trials

Inclusion Criteria

All RCTs published between 2014 and 2026 that evaluated women diagnosed with PMOS or PCOS were assessed and considered for inclusion in this study. Only RCTs involving adult women diagnosed with PCOS or PMOS and comparing liraglutide and metformin as head-to-head evidence, liraglutide and placebo and metformin and placebo as secondary evidence were included.

Full-text articles published in the English language were assessed and included in this systematic review and meta-analysis.

Exclusion Criteria

Non-RCTs, cohort studies, case-control studies, cross-sectional studies, case series, case reports, in vitro studies, animal experiments, commentaries, conference abstracts, editorials, letters to the editor, expert opinions, and review articles were excluded from the review.

Sample Size Calculation

Sample size estimation was not performed, as this systematic review and meta-analysis aimed to include all eligible RCTs published during the predefined search period that met the inclusion criteria.

Data Synthesis

Data synthesis was conducted on the relative titles. The titles were initially screened based on their abstracts. Thereafter, the full texts of the examined RCT titles that met the eligibility requirements were considered for the final selection. All articles were screened independently by two reviewers, KM and IB. Any discrepancies were resolved through discussion and consensus, and in consultation with the third reviewer (JR) when necessary.

Handling of Multi-arm Trials

Multi-arm RCTs were assessed to ensure that participants from the same study were not counted more than once in the same meta-analysis. Where a study included more than one eligible intervention or comparison group, only the relevant arms corresponding to the prespecified comparison of liraglutide versus metformin were included.

Methodology Quality Assessment

The quality assessment of the selected RCTs was assessed independently by two researchers, IB and KM. The Cochrane risk-of-bias tool for randomized trials (RoB 2) was used for quality assessment [13]. The RoB 2 tool is best suited and implemented to assess the domains at low, unclear, and high risk of bias. The risk of bias was assessed on five domains (D1-D5), which were as follows: D1, bias arising from the randomization process; D2, bias due to deviations from intended interventions; D3, bias due to missing outcome data; D4, bias in measurement of the outcome; D5, bias in selection of the reported result. The Risk-Of-Bias VISualization (ROBVIS) tool is a web-based application program that was used to generate the traffic light plots and the weighted bar plots [14].

Data Analysis and Meta-Analysis

The outcomes were analyzed as continuous variables and expressed as mean difference (MD) with standard deviations, and dichotomous variables were expressed as risk ratios (RRs) with 95% confidence intervals (CI). The outcomes were analysed as dichotomous variables and expressed as RRs with 95% CI. Pooled effect estimates were calculated using a fixed-effect model when the statistical heterogeneity among included studies was low (I^2^< 50 %). A random-effects model was applied when substantial heterogeneity was present (I^2^> 50%). Statistical heterogeneity among studies was assessed using the chi-square test. The degree of heterogeneity was assessed by using the I2 statistic [15]. Statistical significance was determined by the Z test, with p-values <0.05 considered statistically significant. Meta-analyses were performed, and forest plots were generated using the latest version of the Review Manager (RevMan Web) application (The Cochrane Collaboration, London, UK) [16].

Results

An extensive literature search yielded a total of 57,313 articles (PubMed: 56,727, Cochrane: 10, TRIP: 84, Google Scholar: 492). Among these, 1,622 were noted as duplicates and were excluded from the initial analysis by EndNote (Clarivate Plc, London, UK); 55,604 records were marked as ineligible based on predetermined criteria. A total of 87 full-text articles were assessed for eligibility. Seven RCTs were finally assessed and were, hence, included for quantitative synthesis for systematic review and meta-analysis [17-23]. PRISMA 2020 guidelines were followed and implemented during the production of this systematic review (Figure 1).

**Figure 1:**
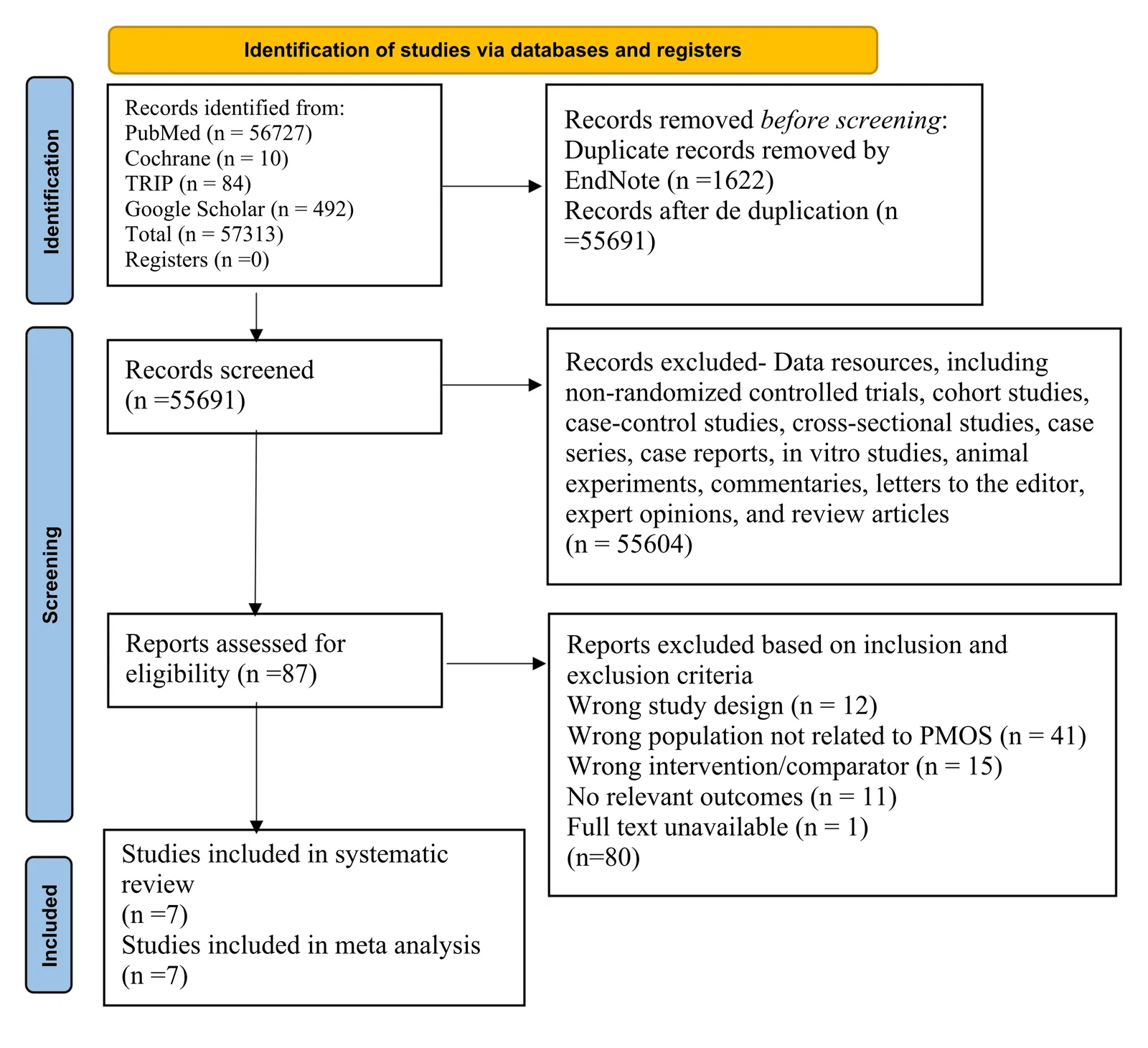
Preferred Reporting Items for Systematic Reviews and Meta-Analyses (PRISMA) 2020 flow diagram illustrating the process of study identification, screening, eligibility assessment and inclusion in the systematic review, including reasons for exclusion at each stage TRIP: Turning Research into Practice; PMOS: polyendocrine metabolic ovarian syndrome

Table 3 shows authors, study year, study design, country, and dose of liraglutide and metformin. All of the included studies were RCTs and have been conducted in Slovenia, Pakistan, Denmark, Finland, and the United States. The studies involved comparison of liraglutide and metformin (two studies of Jensterle et al.,2015 [17]), liraglutide and placebo (Nylander et al.,2017 [20] and Elkind-Hirsch et al.,2022 [23]), and metformin and placebo (Zahra et al., 2017 [19]; Lingaiah et al., 2019 [21]; and Heidari et al.,2019 [22]). The dose of drugs used for liraglutide ranged from 0.6 mg to 2000 mg, and the drug dose for metformin ranged from 0.6 mg to 2000 mg.

**Table 3:** Sample size, country, arms, and dose of liraglutide, metformin and placebo in various RCTs on PMOS PMOS: polyendocrine metabolic ovarian syndrome; RCT: randomized controlled trial; BID: twice a day; PO: per oral; QD: once a day; SC: subcutaneous

Author, study year	Study design	Sample size	Country	Intervention	Control	Remarks	Dose of the intervention group	Dose of the control group
Jensterle et al.,2015 [17]	RCT	32	Slovenia	Liraglutide N=14	Metformin N=14	Four dropouts due to adverse effects	0.6 mg injected SC once per day and increased to 1.2 mg/day after one week	Dose of 500 mg once a day and increased by 500 mg every three days up to 1000 mg BID.
Jensterle et al., 2015 [18]	RCT	41	Slovenia	Liraglutide N=14	Metformin N=13	Roflumilast N=14	1.2 mg QD SC	1000 mg BID PO
Zahra et al., 2017 [19]	RCT	40	Pakistan	Metformin N=20	Placebo N=20	-	500 mg TDS	500 mg TDS?
Nylander et al., 2017 [20]	RCT	72	Denmark	Liraglutide N=48	Placebo N=24	-	0.6 mg the first week, 1.2 mg the second week, and 1.8 mg for 26 weeks	0.6 mg the first week, 1.2 mg the second week, and 1.8 mg for 26 weeks
Lingaiah et al., 2019 [21]	RCT	118	Finland	Metformin N=57	Placebo N=61	Metformin: non-obese (n=40), obese (n=17); Placebo: non-obese (n=27), obese (n=34)	2000 mg	2000 mg
Heidari et al., 2019 [22]	RCT	48	United States	Metformin N=29	Placebo N=13	Six did not complete the trial	1500 mg	1500 mg
Elkind-Hirsch et al.,2022 [23]	RCT	82	United States	Liraglutide N=55	Placebo N=27	-	3 mg	3 mg

Table 4 depicts the age and HOMA-IR of people living with PMOS on liraglutide and metformin. The age for the intervention group ranged from 19.7 to 40.2 years, and the age of the control group ranged from 20.7 to 38.6 years. The HOMA-IR of the intervention group ranged from 0 to 8 for baseline and from 0.8 to 4.7 after treatment, compared to the control group that ranged from 2.1 to 6.4 at baseline and from 0.6 to 6.5 after therapy.

**Table 4:** Age and Homeostatic Model Assessment for Insulin Resistance (HOMA-IR) score of women living with polyendocrine metabolic ovarian syndrome NA: not available

Author, study year	Age (years)		HOMA-IR score			
	Interventional group	Control group	Interventional group baseline	Interventional group after treatment	Control group baseline	Control group after treatment
Jensterle et al., 2015 [17]	29.5 ± 7.7	25.3 ± 5.2	4±4	3.9 ± 2.1	2.7 ± 1.7	2.8 ± 1.4
Jensterle et al., 2015 [18]	30.7 ± 7.9	30.7 ± 7.9	3.8 ± 2.8	3.2 ± 2.4	3.3 ± 1.2	2.5 ± 1.9
Zahra et al., 2017 [19]	25.8 ± 6.1	27.0 ± 6.3	4.2 ± 2.3	3.5 ± 2.3	4.6 ± 1.8	4.8 ± 1.7
Nylander et al., 2017 [20]	30.1 ± 5.5	28.2 ± 3.4	2.29	NA	2.42	NA
Lingaiah et al., 2019 [21]	28.8 ± 3.8	27.3 ± 5.0	4.2	2.8	3.4	3.6
Heidari et al., 2019 [22]	32.4 ± 7.5	33.1 ± 5.9	3.2	2.1	2.1	3.6
Elkind-Hirsch et al., 2022 [23]	31 +/- 0.8	32 +/- 1.1	4.8 ± 0.6	4.1 ± 0.6	5.1 ± 1.0	5.2 ± 1.1

Table 5 shows the frequency of menstrual cycles (number per year) for both the intervention and control groups and the BMI (kg/m²) for the intervention as well as the control group (baseline vs. after-therapy values). The frequency of menstrual cycles ranged from 0.44 to 9.05 cycles per year for the intervention group and from 0.72 to 13.7 cycles per year for the control group. The BMI for the intervention group ranged from 20.2 to 46.9 kg/m² at baseline and ranged from 19.6 to 45.6 kg/m² after therapy. The BMI for the control group ranged from 19.7 to 46.7 kg/m² at baseline and from 20 to 45.8 kg/m² after therapy.

**Table 5:** Frequency of menstrual cycles and BMI of women living with polyendocrine metabolic ovarian syndrome on liraglutide and metformin NA: not available

Author, study year	Frequency of menstrual cycle (number per year)		BMI (kg/m^2^)			
	Interventional group (liraglutide)	Control group (metformin)	Baseline for the interventional group	After therapy for the interventional group	Baseline for the control group	After therapy for the control group
Jensterle et al.,2015 [17]	5.4 ± 4	8.9 ± 4.8	41.6 ± 5.3	40.5 ± 5.1	37.4 ± 6.4	36.5 ± 6.3
Jensterle et al., 2015 [18]	0.74 ± 0.30	0.92 ± 0.20	36.7 ± 5.6	35.6 ± 5.8	39.4 ± 6.9	39.3 ± 7.0
Zahra et al., 2017 [19]	NA	NA	26.7 ± 6.5	25.3 ± 5.7	29.6 ± 9.9	29.7 ± 9.7
Nylander et al., 2017 [20]	NA	NA	33.3 ± 5.1	NA	33.3 ± 4.6	NA
Lingaiah et al., 2019 [21]	NA	NA	33.4 ± 4.3	32.9 ± 4.4	33.3 ± 4.4	33.3 ± 4.5
Heidari et al., 2019 [22]	NA	NA	37.1 ± 9.1	36.2 ± 10.3	38.4 ± 8.3	37.7 ± 8.1
Elkind-Hirsch et al., 2022 [23]	8.65 ± 0.4	4.8 ± 0.65	41.6 +/- 0.9	43.9 +/- 1.5	41.6 +/- 1.1	43.9 +/- 1.7

Table 6 represents the glucose and insulin levels of women living with PMOS on liraglutide and metformin. The glucose level for the intervention group ranged from 4 to 9.48 mmol/l as baseline and from 2.8 to 9.35 mmol/l after therapy. The insulin level for both the intervention and control groups is given in the table.

**Table 6:** Glucose and insulin levels of women living with polyendocrine metabolic ovarian syndrome on liraglutide and metformin NA: not available

Author, study year	Glucose level		Insulin level	
	Interventional group	Control group	Interventional group	Control group
Jensterle et al., 2015 [17]	7 ± 1.9 (mmol/l)	7.9 ± 2.4 (mmol/l)	98.6 ± 95.3 (pmol/l)	112.4 ± 74.3 (pmol/l)
Jensterle et al., 2015 [18]	4.7 ± 0.7 (mmol/l)	4.4 ± 1.6 (mmol/l)	14.5 ± 9.4 (mU/L)	15.5 ± 6.0 (mU/L)
Zahra et al., 2017 [19]	5.60 ± 0.29 (mmol/l)	5.87 ± 0.34 (mmol/l)	14.1 ± 9.3 (IU/L)	18.6 ± 6.4 (IU/L)
Nylander et al., 2017 [20]	NA	NA	NA	NA
Lingaiah et al., 2019 [21]	5.1 ± 0.3 (mmol/l)	5.3 ± 0.3 (mmol/l)	12.1 ± 5.9 (mmol/l)	15.0 ± 7.9 (mmol/l)
Heidari et al.,2019 [22]	7.91 ± 1.57 (mmol/l)	7.58 ± 1.77 (mmol/l)	11.0 ± 5.0 (µIU/ml)	11.7 ± 6.2 (µIU/ml)
Elkind-Hirsch et al.,2022 [23]	5.33 ± 0.09 (mmol/l)	5.28 ± 0.13 (mmol/l)	NA	NA

Table 7 lists adverse drug reactions for liraglutide and metformin. Liraglutide was found to cause nausea, diarrhea, vomiting, constipation, stomachache, heartburn, headache, and dizziness. Liraglutide showed more constipation, gastroenteritis, epigastric pain, cholecystectomy, hypotension, tachycardia, and syncope compared to metformin. Placebo showed an increase in upper respiratory tract infections.

**Table 7:** Various adverse drug reactions of liraglutide and metformin in women with polyendocrine metabolic ovarian syndrome LIRA: liraglutide; MET: metformin; PL: placebo; GI: gastrointestinal; NA: not available

Author, study year	Adverse drug reaction	
	LIRA n (%)/ MET n (%)	MET n (%)/ PL n (%)
Jensterle et al., 2015 [17]	LIRA: Nausea 8/14 (57.1)	MET: GI problems, nausea and/or diarrhea 9/14 (64.3)
	LIRA: Diarrhea 3/14 (21.4)	-
	LIRA: Vomiting 1/14 (7.1)	-
	LIRA: Constipation 1/14 (7.1)	-
	LIRA: Stomach ache 1/14(7.1)	-
	LIRA: Heartburn 1/14 (7.1)	-
	LIRA: Headache 2/14 (14.3)	-
	LIRA: Dizziness 4/14 (28.6)	-
Jensterle et al., 2015 [18]	LIRA: Nausea 4/14 (28.6)	MET: Diarrhea 4/14 (28.6)
	LIRA: Constipation 2/14 (14.3)	-
	LIRA: Diarrhea 1/14 (7.1)	-
	LIRA: Headache 1/14 (7.1), Insomnia1/14 (7.1)	-
Zahra et al., 2017 [19]	MET: Menstrual irregularities	PL: Menstrual irregularities
Nylander et al., 2017 [20]	LIRA: Nausea 37/48 (78.7)	PL: Nausea 3/24 (13.0)
	LIRA: Vomiting 5/48 (10.6)	PL: Vomiting 0 (0)
	LIRA: Ructus/heartburn 8/48 (17.0)	PL: Ructus/heartburn 0 (0)
	LIRA: Diarrhea 5/48 (10.6)	PL: Diarrhea 1/24 (4.4)
	LIRA: Constipation 12/48 ( 25.5)	PL: Constipation 0 (0)
	LIRA: Gastroenteritis 5/48 (10.6)	PL: Gastroenteritis 2/24 (8.7)
	LIRA: Epigastric pain 8/48 (17.0)	PL: Epigastric pain 0 (0)
	LIRA: Gallstone-related pain 3/48 (6.4)	PL: Gallstone-related pain 1/24 (4.4)
	LIRA: Cholecystectomy 2/48 (4.3)	PL: Cholecystectomy 0 (0)
	LIRA: Hypotension 1/48 (2.1)	PL: Hypotension 0 (0)
	LIRA: Tachycardia 1/48 (2.1)	PL: Tachycardia 0 (0)
	LIRA: Syncope 1/48 (2.1)	PL: Syncope 0 (0)
	LIRA: Dizziness 4/48 (8.5)	PL: Dizziness 0 (0)
	LIRA: Headache 0/48 (0)	PL: Headache 3/24 (13)
	LIRA: Upper respiratory tract infection 7/48 (14.9)	PL: Upper respiratory tract infection 4/24 (17.4)
	LIRA: Urinary tract infection 2/48 (4.3)	PL: Urinary tract infection 0(0)
	LIRA: Hair loss 1/48 ( 2.1)	PL: Hair loss 0(0)
Lingaiah et al., 2019 [21]	NA	NA
Heidari et al., 2019 [22]	NA	NA
Elkind-Hirsch et al., 2022 [23]	NA	NA

Risk of Bias Assessment

The selected articles were critically appraised. Figure 2 illustrates the results. The results of the assessment across the five domains showed that bias due to the randomization process was low risk (100%), bias from intended interventions was low risk (100%), bias due to missing outcome data was low risk (100%), bias due to measurement of outcome was low risk, with some concerns and high risks. Bias due to selection of reported results was low risk, with some concerns and high risks. Overall, the risk of bias was low risk, with some concerns and high risks. The RobVis tool was used to generate the traffic light plot (Figure 2a), and the summary/weighted bar plot (Figure 2b) depicts the risk of bias.

**Figure 2:**
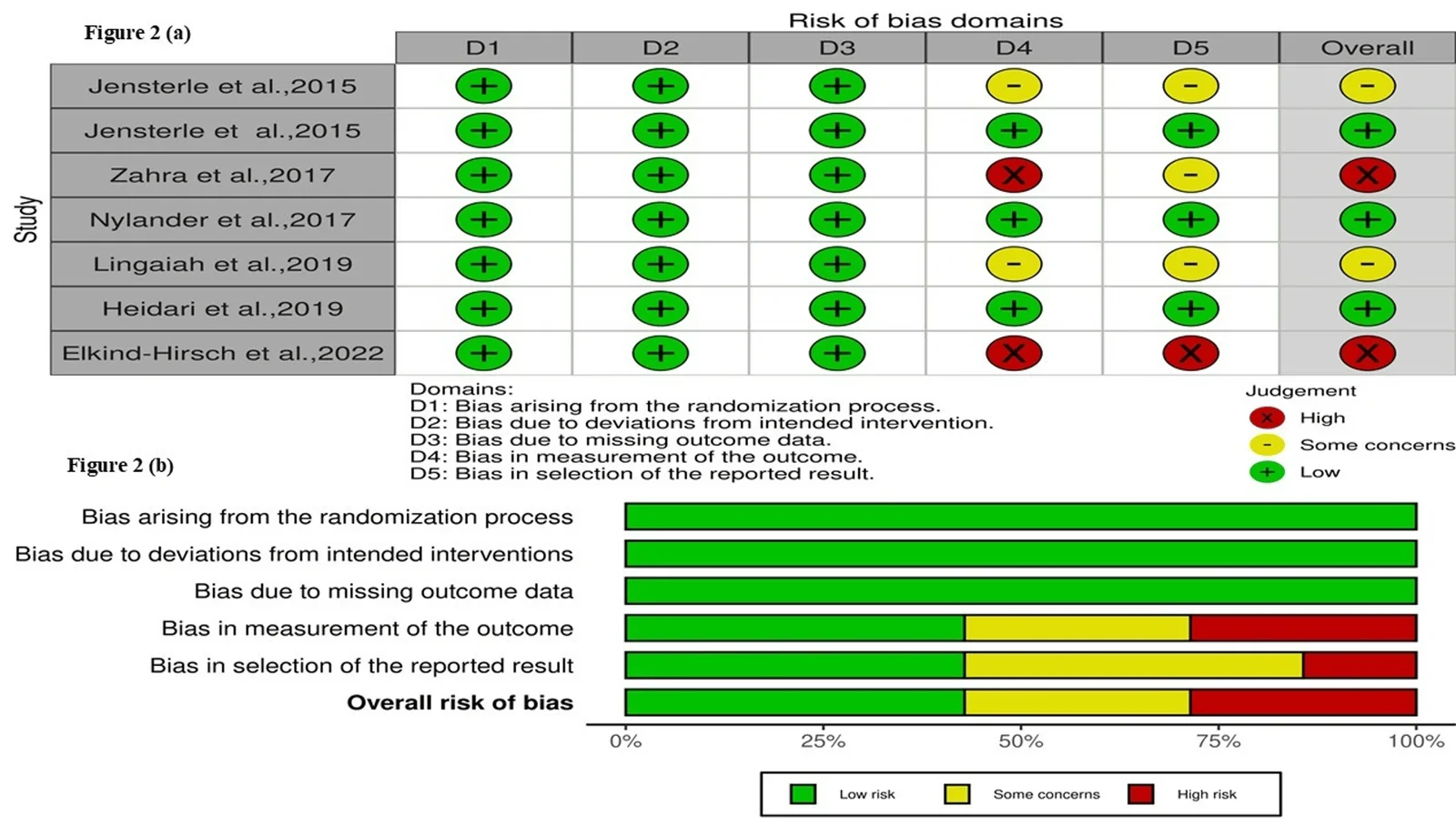
Risk of bias assessments of RCTs a: traffic light plot; b: summary of risk of bias for RCTs (weighted bar plots) RCTs: randomized controlled trials Sources: [17-23]

Age

Head-to-head evidence between liraglutide and metformin: Baseline age was comparable between liraglutide and metformin groups, with no significant difference observed (MD= 2.48 years, 95% CI: -1.26-6.22, P=0.19), indicating adequate baseline similarity between treatment groups (Figure 3a).

**Figure 3:**
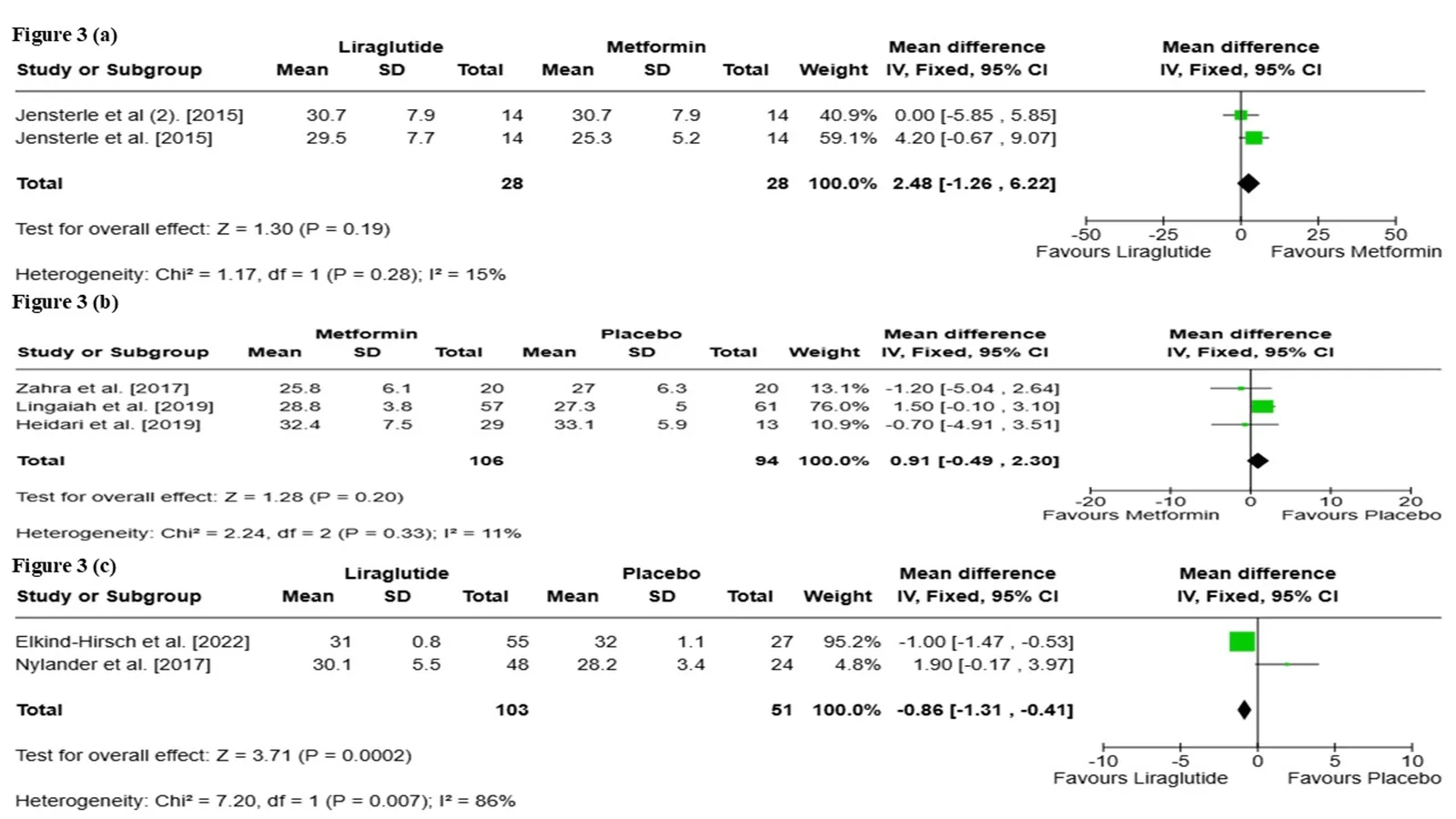
Forest plots of ages of patients with polyendocrine metabolic ovarian syndrome a: liraglutide versus metformin; b: metformin versus placebo; c: liraglutide versus placebo

Placebo-controlled evidence: For the metformin and placebo groups, there was no significant difference in baseline age between participants receiving metformin and placebo (MD = -0.91 years, 95% CI: -0.49-2.30, P = 0.20). The analysis showed low evidence of heterogeneity among studies (I^2^=11%) (Figure 3b). For liraglutide and placebo groups, a pooled analysis of the baseline age showed a statistical difference between liraglutide and placebo groups (MD=-0.86 years, 95% CI: -1.31 to -0.41, p=0.0002). However, substantial heterogeneity was observed among the studies (I²=86%). As age is a baseline characteristic rather than a treatment outcome, this difference was considered a potential baseline imbalance (Figure 3c).

HOMA-IR 

Head-to-head evidence between liraglutide and metformin: Figure 4 depicts the meta-analysis involving 28 participants in the liraglutide group and 27 participants in the metformin group, indicating HOMA-IR values. A higher HOMA-IR value indicates greater insulin resistance. The pooled result favored metformin but showed no statistical difference between liraglutide and metformin (MD=0.94, 95% CI: -0.09-1.97, P=0.07). No heterogeneity was observed in the fixed model (I²=0%, chi-square=0.14, p=0.71).

**Figure 4:**
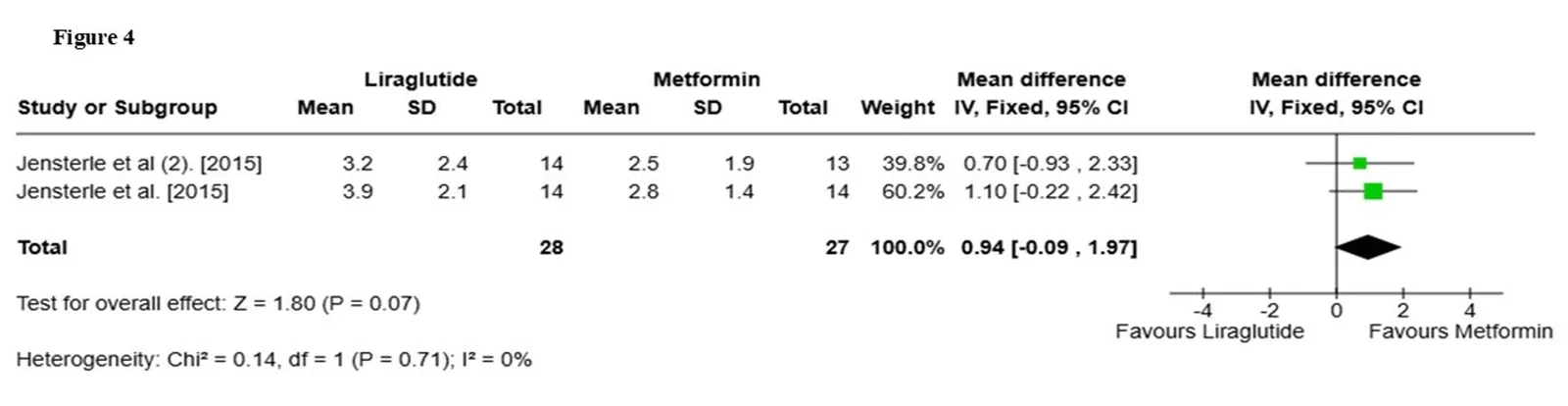
Forest plot of Homeostatic Model Assessment for Insulin Resistance (HOMA-IR) for patients with polyendocrine metabolic ovarian syndrome

BMI

Head-to-head evidence between liraglutide and metformin: Figure 5a depicts a random-effects meta-analysis that found no statistically significant difference in BMI between liraglutide and metformin (MD=0.25, 95% CI: -7.30-7.79, P=0.95, I^2^=82%, p=0.02). 

**Figure 5:**
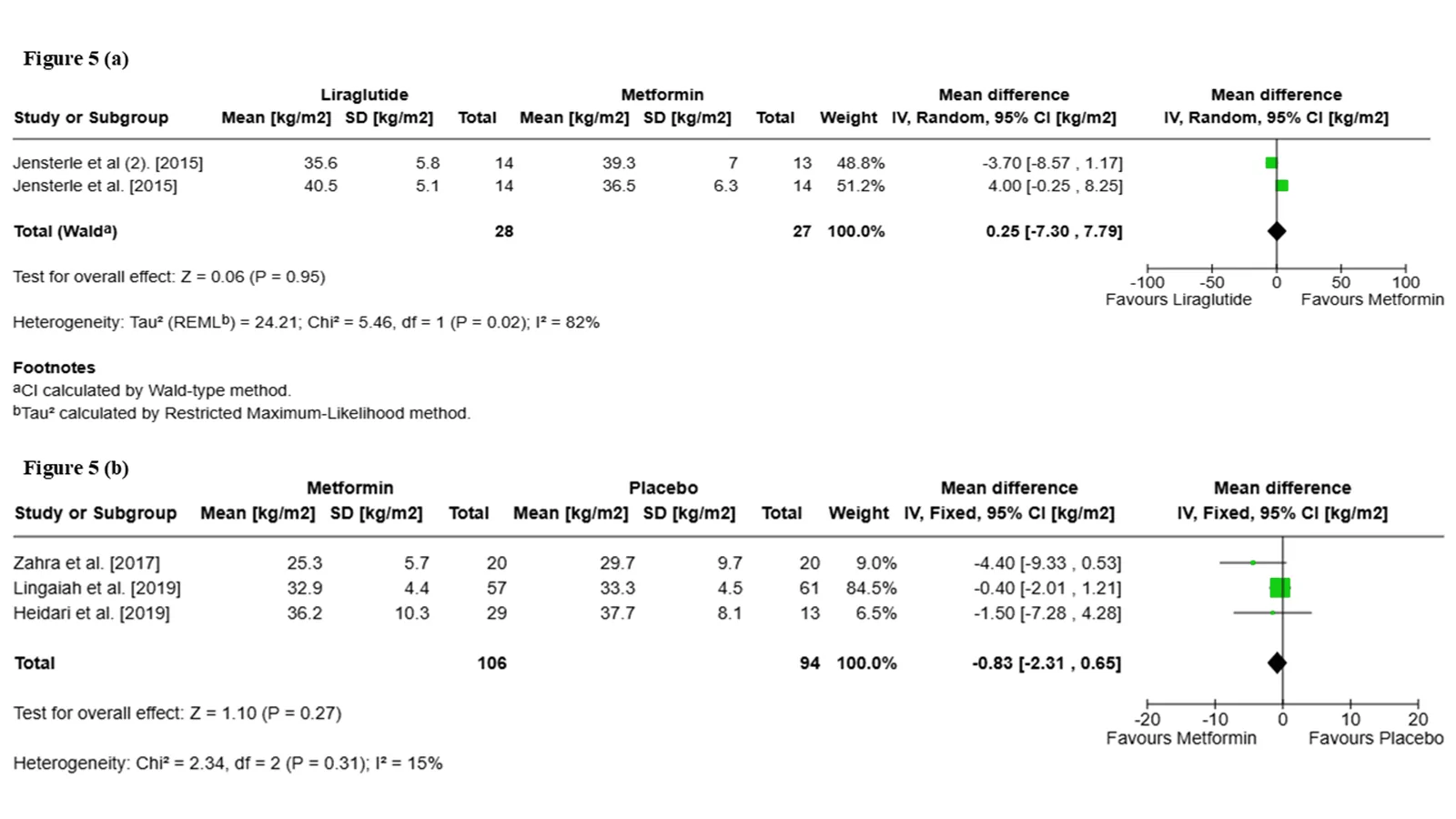
Forest plots of BMI of patients with polyendocrine metabolic ovarian syndrome a: liraglutide versus metformin; b: metformin versus placebo

Placebo-controlled evidence: A fixed-effect model forest plot suggests that metformin was associated with a small fraction reduction in BMI as compared to placebo, although it showed no significant difference (MD=-0.83 kg/m^2^, 95 CI: -2.31- 0.65, P=0.27). The analysis demonstrated low heterogeneity among the included studies (I^2^=15%, p=0.31) Figure 5b.

Frequency of Menstrual Cycle (Number Per Year)

Head-to-head evidence between liraglutide and metformin: A random effects meta-analysis demonstrated no significant difference between liraglutide and metformin (MD= -1.42, 95% CI: -4.57-1.73, P=0.38). Although the pooled effect favored metformin due to a higher frequency of the menstrual cycle. A substantial heterogeneity was observed (I^2^=75%) (Figure 6).

**Figure 6:**
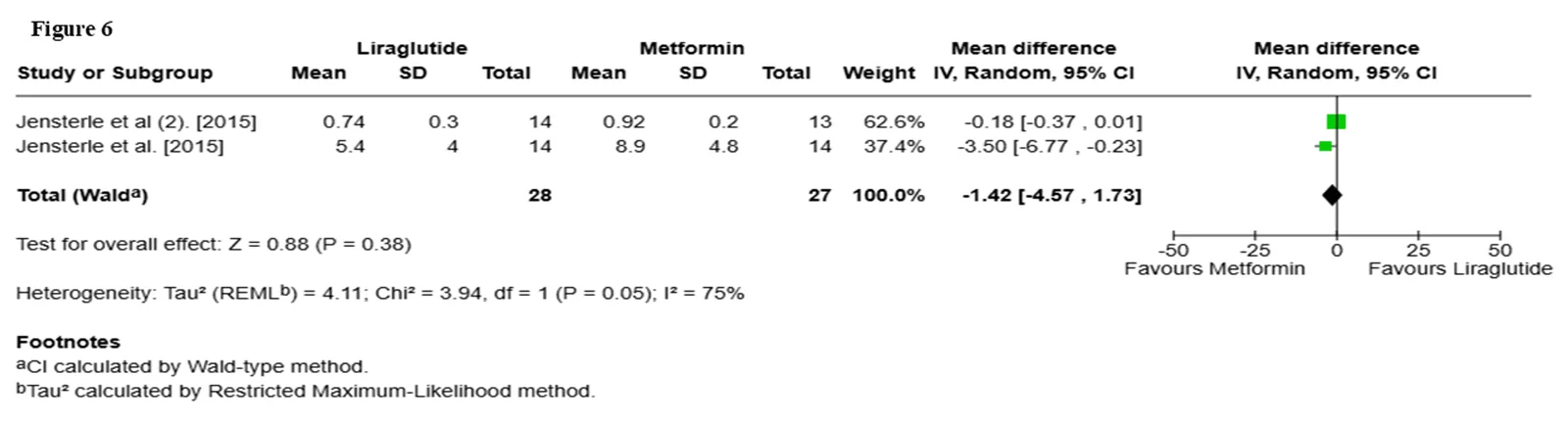
Forest plot of frequency of menstrual cycle/number per year of patients with polyendocrine metabolic ovarian syndrome, liraglutide versus metformin

Glucose Level

Head-to-head evidence between liraglutide and metformin: A fixed-effect meta-analysis demonstrated no significant difference in fasting glucose levels between liraglutide and metformin (MD=-0.01 mmol/l, 95 % CI: - 0.82-0.80, P=0.98). Low heterogeneity was observed across studies (I^2^=37%, p=0.21).

Placebo-controlled evidence​​​​: A fixed-effect meta-analysis involving 200 participants (metformin: 200, placebo: 94) significantly reduced glucose levels compared to placebo (MD= -0.21 mmol/l, 95% CI: -0.31 to-0.12, p<0.0001). The effect was consistent across studies, with no observed heterogeneity (I^2^=0%, p-0.53) (Figure 7).

**Figure 7:**
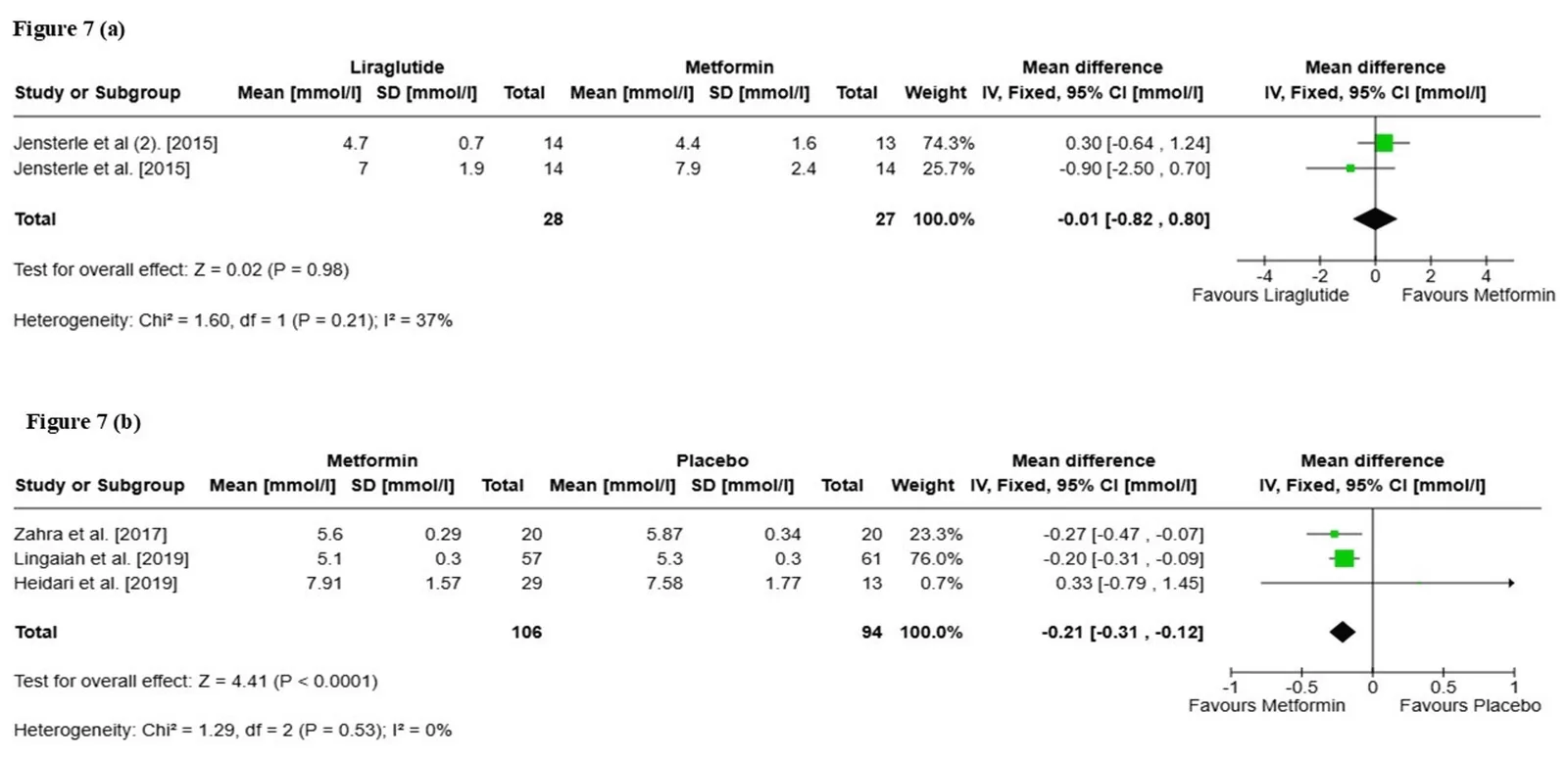
Forest plots of glucose levels of patients with polyendocrine metabolic ovarian syndrome a: liraglutide versus metformin; b: metformin versus placebo

Insulin Level

Head-to-head evidence between liraglutide and metformin: Figure 8a demonstrates that there was no significant difference in fasting insulin levels between liraglutide and metformin (MD=-1.11 Mu/L, 95% CI: -6.99-4.77, p=0.71). No heterogeneity was observed among included studies (I^2^=0%, p=0.69). 

**Figure 8:**
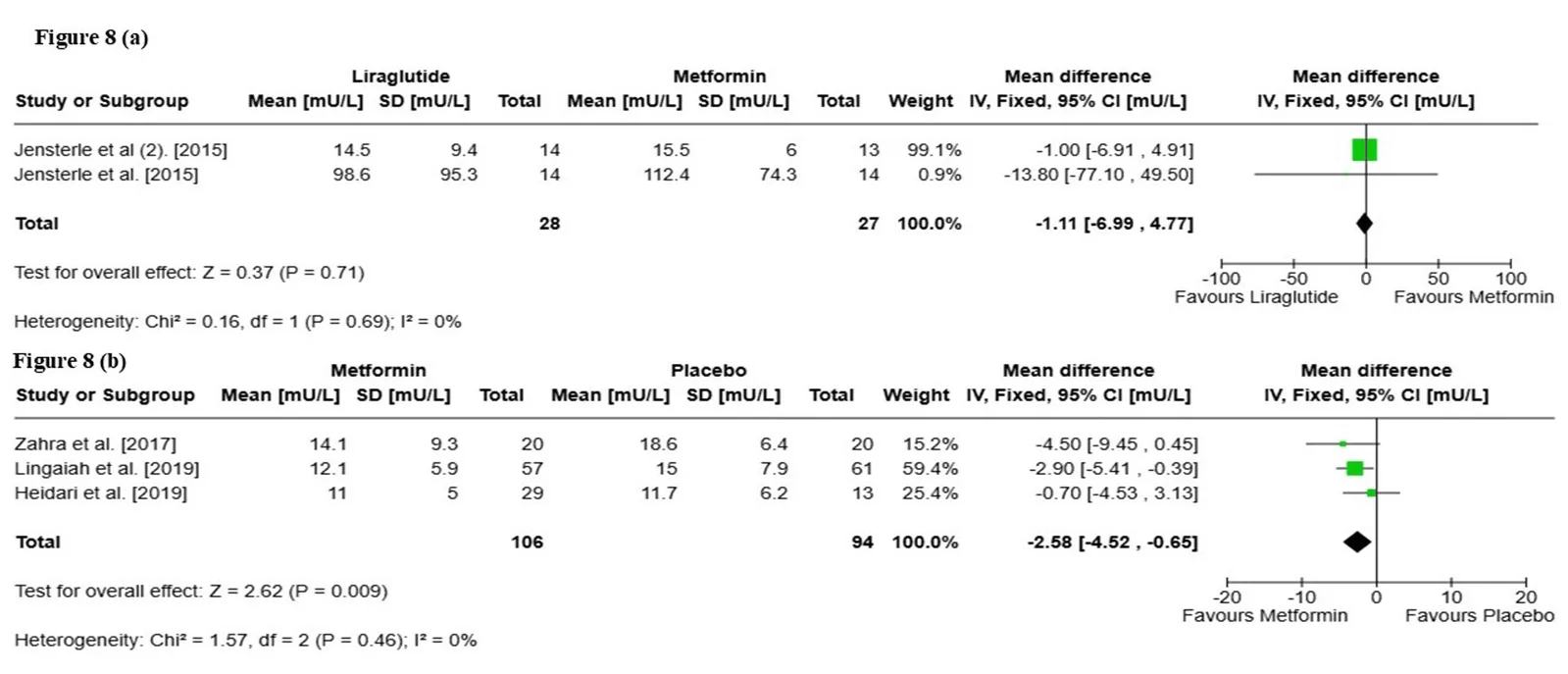
Forest plots of insulin level of patients with polyendocrine metabolic ovarian syndrome a: liraglutide versus metformin; b: metformin versus placebo

Placebo-controlled evidence: A fixed-effect meta-analysis including 220 participants (metformin: 106, placebo: 94) demonstrated that metformin significantly reduced insulin levels as compared to placebo (MD=-2.58 Mu/l, 95% CI: -4.52 to-0.65, P=0.009). No significant heterogeneity was detected among studies (I^2^=0%, p=0.46), indicating consistent treatment effects across the included trials. The reduction of 2.58 Mu/l insulin levels supports the insulin-sensitizing effect of metformin Figure 8b.

Adverse Drug Reactions

Nausea (head-to-head evidence between liraglutide and metformin): Figure 9a represents the pooled analysis demonstrated no significant difference in the risk of nausea (RR=0.91, 95% CI: 0.53-1.55, P=0.73. Although the effect estimates numerically favored liraglutide, no heterogeneity was observed among studies (I^2^=0%, p=0.86).

**Figure 9:**
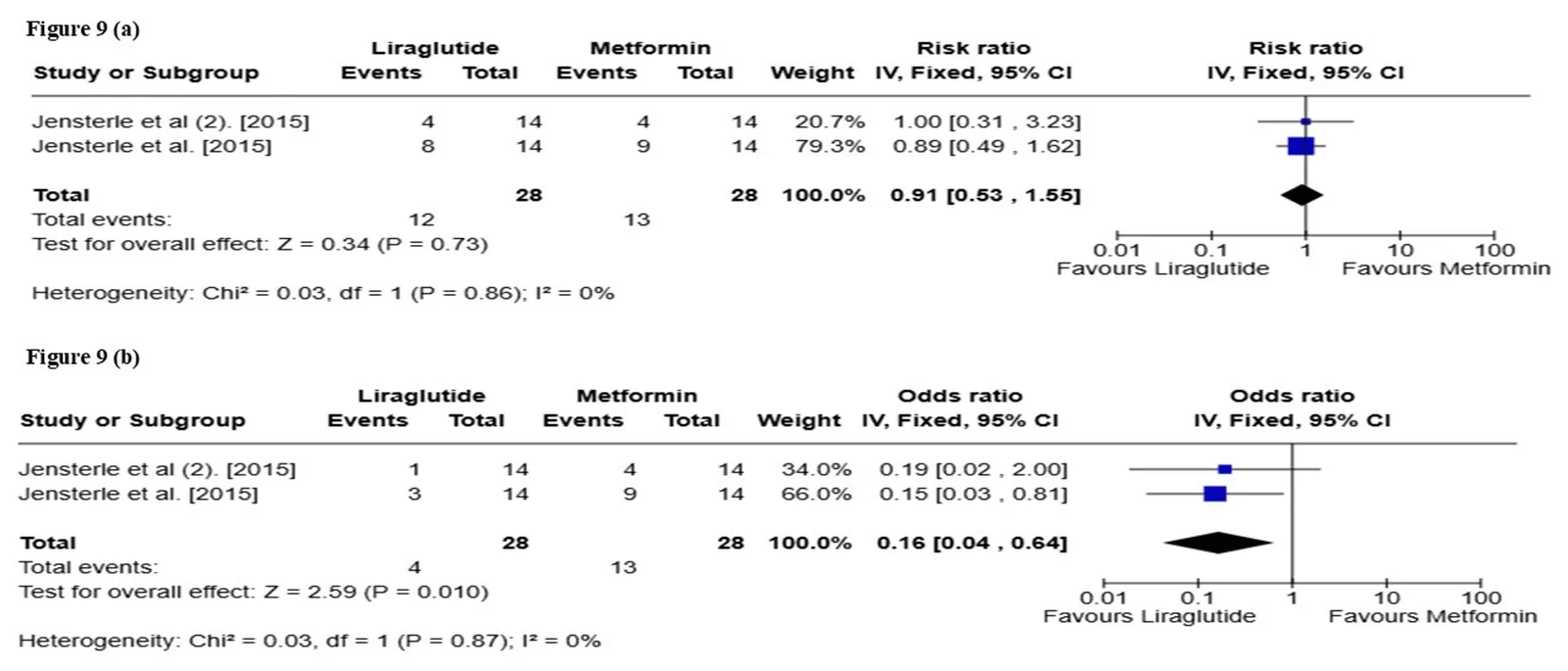
Forest plots of adverse effects in patients with polyendocrine metabolic ovarian syndrome a: nausea, liraglutide versus metformin; b: diarrhea, liraglutide versus metformin

Diarrhea (head-to-head evidence between liraglutide and metformin): The incidence of diarrhea was significantly lower in the liraglutide group compared to metformin. The pooled analysis demonstrated a reduced risk of diarrhea with liraglutide (RR=0.31, 95% CI: 0.12-0.81, P=0.02), suggesting a 69% relative risk reduction as compared to metformin. No heterogeneity was observed (chi-square = 0.03, df=1, p=0.81, I^2^=0%) (Figure 9b).

Discussion

Principal Findings: Direct Head-to-Head Liraglutide Versus Metformin Evidence

This systematic review and meta-analysis evaluated the comparative efficacy and tolerability of liraglutide and metformin in women with PMOS, formerly known as PCOS. The principal finding was that the available randomized evidence did not demonstrate a statistically significant difference between liraglutide and metformin in BMI, insulin resistance measured using HOMA-IR, fasting glucose, fasting insulin, or menstrual-cycle frequency. The risk of nausea was also comparable between the two treatments. However, diarrhea occurred significantly less frequently with liraglutide than with metformin. Collectively, these findings suggest broadly comparable short-term effects of liraglutide and metformin across several metabolic and reproductive outcomes, with a potential difference in gastrointestinal tolerability.

Importantly, these findings are based primarily on direct head-to-head comparisons between liraglutide and metformin. The absence of statistically significant differences should not be interpreted as evidence of therapeutic equivalence because the comparisons were based on a limited number of participants and, for some outcomes, had wide confidence intervals and substantial heterogeneity.

Contextual Interpretation of Metabolic Outcomes

These findings should be interpreted within the broader understanding of PMOS as a lifelong, multisystem endocrine and metabolic condition rather than an exclusively ovarian disorder. Insulin resistance, compensatory hyperinsulinaemia, excess adiposity, hyperandrogenism and ovulatory dysfunction interact closely in its pathophysiology, supporting the assessment of metabolic, anthropometric and reproductive outcomes together when evaluating treatment efficacy [1,2].

Lifestyle intervention remains the foundation of management, while pharmacological treatment should be selected according to the patient’s metabolic risk, body weight, reproductive priorities, treatment preferences and tolerance [1,3]. Current international guidance primarily supports metformin for metabolic abnormalities and recognizes GLP-1 RAs, including liraglutide, as options for weight management in adults with PMOS and excess weight [3].

Insulin Resistance: HOMA-IR

The pooled direct head-to-head comparison showed no significant difference in HOMA-IR between liraglutide and metformin, although the direction of effect numerically favoured metformin. This finding differs from some earlier meta-analyses in which GLP-1 RAs produced greater reductions in HOMA-IR than metformin [7,10,24,25]. Nevertheless, it is consistent with other evidence demonstrating no clear difference between GLP-1 RAs and metformin for fasting insulin or HOMA-IR, particularly when analyses were restricted to small head-to-head trials [4,9]. These apparently conflicting findings may partly reflect differences in the drugs pooled within earlier reviews. Several previous meta-analyses combined liraglutide with exenatide or other GLP-1 RAs, despite potential differences in their pharmacokinetic properties, dosing schedules and metabolic effects [4,7,9,10,24,25]. Some also combined monotherapy with liraglutide-metformin combination therapy or included placebo and standard-care comparators. Such approaches increase statistical power but make it difficult to determine the drug-specific effect of liraglutide relative to metformin.

The short duration of the direct comparative trials may also have limited the measurable effect on insulin resistance. In an early randomized study, both liraglutide and metformin reduced weight-related parameters, but neither produced a clear improvement in HOMA-IR over 12 weeks [17]. Similarly, another short-term trial found no statistically significant between-group difference in HOMA-IR despite improvements in glucose tolerance with liraglutide [18]. By contrast, longer placebo-controlled treatment with liraglutide 3 mg improved HOMA-IR, glucose regulation and other measures of insulin sensitivity over 32 weeks [23]. Therefore, the metabolic response to liraglutide may depend on treatment duration, dose, baseline obesity and severity of insulin resistance. The liraglutide dose used in several direct comparative trials was 1.2 mg daily, whereas the more pronounced metabolic and weight-related effects in later research were observed with 3 mg daily [17,18,23].

Fasting Glucose and Fasting Insulin: Direct Head-to-Head Comparative Evidence

The absence of a significant difference in fasting glucose between liraglutide and metformin is biologically plausible because both medications influence glucose regulation through different but potentially overlapping pathways. Metformin reduces hepatic glucose production, enhances peripheral glucose utilization, and improves insulin sensitivity, primarily through pathways involving AMP-activated protein kinase [5,6].

Similarly, the direct comparison showed no statistically significant difference in fasting insulin between liraglutide and metformin. Previous syntheses have produced mixed conclusions regarding the relative effect of GLP-1 RAs on fasting insulin. Some reported improvements in fasting insulin and HOMA-IR [8,24,25], whereas others found no consistent effect after accounting for study quality, comparator, and heterogeneity [4,9]. Variations in baseline insulin resistance may be especially important. Women with severe obesity or higher HOMA-IR may experience a greater metabolic response to liraglutide than those with less severe metabolic dysfunction [17]. Pooling participants across different PMOS phenotypes may consequently dilute clinically meaningful subgroup effects.

BMI and Weight-Related Outcomes

No statistically significant difference in BMI was detected between liraglutide and metformin. Although the pooled estimate marginally favoured metformin, the CI was extremely wide and substantial heterogeneity was present. Accordingly, the finding does not establish that metformin is superior or that both drugs have equivalent effects on body weight. Rather, it indicates considerable uncertainty in the available direct comparative evidence. The heterogeneity may be attributable to variation in baseline BMI and metabolic severity, differences in liraglutide and metformin doses, treatment duration, adherence, lifestyle co-interventions and methods used to calculate changes in BMI.

This result differs from several meta-analyses reporting greater reductions in BMI, body weight and waist circumference with GLP-1 RAs than with metformin [7,24,25]. A pilot randomized study also found liraglutide to be superior to metformin for reducing body weight, BMI, waist circumference and visceral adiposity over 12 weeks [18]. However, another direct randomized trial found that both treatments significantly reduced BMI, weight, waist circumference and total fat mass, without a significant overall difference between the treatment groups [17]. In that study, liraglutide appeared more effective in a post hoc subgroup with severe obesity and greater insulin resistance, suggesting that baseline metabolic phenotype may modify the response [17]. Furthermore, the phase 3 placebo-controlled trial of liraglutide 3 mg demonstrated clinically meaningful weight loss, reductions in central adiposity and preservation of lean mass [23]. These findings confirm that liraglutide can produce substantial weight loss, but they do not necessarily prove superiority over adequately dosed metformin in every population.

The difference between the present findings and previous meta-analyses may also be explained by comparator selection. Liraglutide generally demonstrates larger weight reductions when compared to placebos than when compared with an active treatment such as metformin [4,23-25]. Some previous reviews pooled various GLP-1 RAs and treatment combinations, potentially producing larger effect estimates than an analysis focused specifically on liraglutide monotherapy versus metformin [7,8,24,25]. In addition, metformin is not considered a primary anti-obesity medication, but it can produce modest weight reduction, particularly when combined with lifestyle modification [1,5,6]. Thus, a small difference between two active interventions may be difficult to demonstrate in short and underpowered trials.

Menstrual Cycle Frequency

The pooled direct comparison analysis did not identify a significant difference in the annual frequency of menstrual cycles between liraglutide and metformin. This finding is consistent with earlier comparative evidence showing no clear superiority of GLP-1 RAs over metformin for menstrual frequency [4,7,9,17]. Nevertheless, some studies and meta-analyses have reported improved menstrual regularity after GLP-1 RA treatment, particularly with longer treatment durations [8,23]. One meta-analysis found that GLP-1 RAs improved menstrual cyclicity and natural pregnancy rates, although substantial heterogeneity and variation in concomitant metformin use reduced certainty in these findings [8]. The phase 3 liraglutide study also reported a marked increase in menstrual frequency after 32 weeks of treatment [23].

The effect of liraglutide on menstrual function is likely to be indirect and may require sufficient time and weight reduction to become apparent. Reduced adiposity and hyperinsulinaemia may lower ovarian androgen production, increase sex hormone-binding globulin and improve hypothalamic-pituitary-ovarian signalling, thereby supporting follicular development and ovulation [1,8]. A 12-week treatment period may therefore be sufficient to detect changes in appetite or weight but too short to reliably evaluate menstrual regularity, ovulation or fertility. Menstrual frequency is also influenced by baseline cycle irregularity, PMOS phenotype, hormonal contraception, fertility treatments and the method used to record menstruation. These factors may explain the substantial heterogeneity observed in the present analysis. Moreover, menstrual regularity is not equivalent to ovulation, pregnancy or live birth; future studies should assess these outcomes separately using standardized definitions. Although liraglutide and metformin have shown promising results in improving menstrual-cycle frequency, a recent meta-analysis by Banerjee et al. reported that letrozole remains the drug of choice for inducing ovulation and achieving pregnancy in women with PMOS, supported by high- to moderate-certainty evidence [26]. 

Gastrointestinal Tolerability

The safety findings provide an important practical distinction between the two treatments. Nausea did not differ significantly between liraglutide and metformin, whereas liraglutide was associated with a significantly lower risk of diarrhea. Both medications commonly cause gastrointestinal symptoms, but their patterns differ. Nausea, vomiting, abdominal discomfort, and constipation are frequently reported with GLP-1 RAs, especially during dose escalation [4,7,23,25]. Diarrhea and general gastrointestinal discomfort are common with metformin, particularly during treatment initiation or rapid dose escalation [5,6]. The lower risk of diarrhea with liraglutide in the present analysis is therefore clinically plausible and may be relevant for patients who discontinue metformin because of persistent bowel-related adverse effects. However, the comparable risk of nausea should be interpreted cautiously because adverse-event analyses were based on few studies and events. Previous meta-analyses have reported a higher incidence of nausea with GLP-1 RAs than with metformin or other controls [7,25]. Differences in titration schedules, liraglutide dose, metformin formulation, reporting thresholds and treatment duration may account for the inconsistency.

Secondary Placebo-Controlled Evidence

Secondary placebo-controlled analyses provide supportive evidence regarding the efficacy of metformin compared with no active treatment, rather than evidence regarding the relative efficacy of metformin versus liraglutide. In these analyses, metformin showed significantly reduced fasting glucose and fasting insulin compared with placebo, although its effect on BMI was not statistically significant.

In the present analysis, metformin significantly reduced fasting glucose compared with placebo, supporting its established glucose-lowering activity in women with PMOS. This is consistent with reviews describing metformin as a reliable metabolic therapy, particularly in women who are overweight and suffer from obesity, insulin resistance or increased risk of dysglycaemia [3,5,6].

Clinical Implications

These findings do not support the routine replacement of metformin with liraglutide for all women with PMOS. Metformin remains an inexpensive, widely available oral treatment with established benefits for glucose regulation, insulin resistance and selected reproductive outcomes [3,5,6]. It may be particularly appropriate when metabolic dysfunction, dysglycaemia, affordability or an oral route of administration is the main consideration. Liraglutide may be more suitable for women with obesity or central adiposity in whom clinically meaningful weight reduction is a major treatment goal, particularly when lifestyle intervention and metformin have been insufficient, or metformin is poorly tolerated [1,3,4,17,18,23].

Treatment selection should nevertheless incorporate the burden of daily injections, gastrointestinal effects, cost, availability and the possibility of weight regain after discontinuation. GLP-1 RAs should not be used during pregnancy, and effective contraception is recommended during treatment because pregnancy-safety data remain insufficient [1,3]. Women actively seeking pregnancy may therefore require a different therapeutic strategy. Metformin may improve menstrual and ovulatory function in selected women, but it is not the preferred first-line pharmacological treatment for ovulation induction; letrozole remains the recommended first-line option for anovulatory infertility when no other infertility factors are present [5,6].

Overall Interpretation and Research Implications

Overall, the evidence suggests that liraglutide and metformin have no demonstrated statistically significant difference across the principal metabolic and reproductive outcomes evaluated in the head-to-head trials, while liraglutide may have a more favourable profile for diarrhoea. The secondary placebo-controlled findings reinforce the established activity of metformin but should not be used to rank metformin against liraglutide.

Future adequately powered, longer duration randomized trials should directly compare standardized doses of liraglutide and metformin and should stratify participants according to baseline obesity, insulin resistance and PMOS phenotype. Outcomes should include standardized measures of weight, insulin resistance, and menstrual regularity, together with systematic assessment of gastrointestinal and other adverse events. Such studies would help determine whether particular metabolic or reproductive phenotypes derive greater benefit from one treatment than the other

Limitations

The major limitation of this systematic review and meta-analysis is the small number of eligible RCTs. Only seven RCTs in women with PMOS met the inclusion criteria. Furthermore, only two RCTs compared liraglutide and metformin directly, and other RCTs compared with placebo. Consequently, head-to-head comparison of liraglutide and metformin remains limited. Future well-designed, adequately powered RCTs directly comparing Liraglutide and metformin in women with PMOS are needed to provide more better evidence regarding their comparative efficacy and safety. Publication bias could not be formally assessed because of the limited number of included studies. 

## Conclusions

The available randomized evidence suggests that liraglutide and metformin have comparable effects on BMI, insulin resistance measured by HOMA-IR, fasting glucose, fasting insulin, and menstrual-cycle frequency in women with PMOS.

Although both agents have demonstrated clinical benefits in the management of PMOS, the current evidence does not demonstrate a statistically significant advantage of liraglutide over metformin for the assessed outcomes. However, the absence of a statistically significant difference cannot be interpreted as evidence of therapeutic equivalence or non-inferiority, nor does it exclude a clinically meaningful benefit of either treatment. Further well-designed, adequately powered RCTs are warranted to establish the comparative effectiveness and long-term safety of these treatments.
